# The global relevance of locally grounded ethnobiology

**DOI:** 10.1186/s13002-024-00693-w

**Published:** 2024-05-18

**Authors:** Álvaro Fernández-Llamazares, Irene Teixidor-Toneu, Chelsey Geralda Armstrong, Julián Caviedes, José Tomás Ibarra, Dana Lepofsky, Alex C. McAlvay, Zsolt Molnár, R. Mónica Moraes, Guillaume Odonne, Melissa R. Poe, Abolfazl Sharifian Bahraman, Nancy J. Turner

**Affiliations:** 1 Departament de Biologia Animal, Biologia Vegetal i Ecologia (BABVE), Universitat Autònoma de Barcelona, Cerdanyola del Vallès, Barcelona, Spain; 2 Institut de Ciència i Tecnologia Ambientals (ICTA-UAB), Universitat Autònoma de Barcelona, Cerdanyola del Vallès, Barcelona, Spain; 3 IMBE, Aix Marseille Univ, Avignon Univ, CNRS, IRD, Marseille, France; 4 Indigenous Studies, Simon Fraser University, Burnaby, BC Canada; 5 ECOS (Ecosystem – Complexity – Society) Co-Laboratory, Center for Local Development (CEDEL) & Center for Intercultural and Indigenous Research (CIIR), Pontificia Universidad Católica de Chile, Villarrica, Chile; 6 Cape Horn International Center for Global Change Studies and Biocultural Conservation (CHIC), Universidad de Magallanes, Puerto Williams, Chile; 7 Faculty of Agriculture and Natural Systems & Center of Applied Ecology and Sustainability (CAPES), Pontificia Universidad Católica de Chile (PUC), Santiago, Chile; 8 Department of Archaeology, Simon Fraser University, Burnaby, BC Canada; 9 Institute of Economic Botany, New York Botanical Garden, Bronx, NY USA; 10 HUN-REN Centre for Ecological Research, Vácrátót, Hungary; 11 Instituto de Ecología, Universidad Mayor de San Andrés, La Paz, Bolivia; 12 Herbario Nacional de Bolivia, La Paz, Bolivia; 13 UAR 3456 LEEISA (Laboratoire Ecologie, Evolution, Interactions Des Systèmes Amazoniens), CNRS, Université de Guyane, IFREMER, Cayenne, France; 14 University of Washington, Seattle, WA USA; 15 Range and Watershed Management Department, Gorgan University of Agricultural Sciences and Natural Resources, Gorgan, Iran; 16 School of Environmental Studies, University of Victoria, Victoria, BC Canada

**Keywords:** Global environmental change, Glocalization, Indigenous and local knowledge, Scalability, Sustainability

## Abstract

While ethnobiology is a discipline that focuses on the local, it has an outstanding, but not yet fully realized potential to address global issues. Part of this unrealized potential is that universalistic approaches often do not fully recognize culturally grounded perspectives and there are multiple challenges with scaling up place-based research. However, scalability is paramount to ensure that the intimate and context-specific diversity of human–environmental relationships and understandings are recognized in global-scale planning and policy development. Here, we identify four pathways to enable the scalability of place-based ethnobiological research from the ground up: local-to-global dialogues, aggregation of published data, multi-sited studies, and geospatial analyses. We also discuss some major challenges and consideration to encourage continuous reflexivity in these endeavours and to ensure that scalability does not contribute to unnecessarily decontextualizing, co-opting, or overwriting the epistemologies of Indigenous Peoples and local communities. As ethnobiology navigates multiple scales of time and space and seeks to increase its breadth, this study shows that the use of deliberately global approaches, when carefully nested within rich field-based and ecological and ethnographically grounded data, can contribute to: (1) upscaling case-specific insights to unveil global patterns and dynamics in the biocultural contexts of Indigenous Peoples and local communities; (2) bringing ethnobiological knowledge into resolutions that can influence global environmental research and policy agendas; and (3) enriching ethnobiology’s field-based ethos with a deliberate global analytical focus.

**Supplementary Information:**

The online version contains supplementary material available at 10.1186/s13002-024-00693-w.

## Introduction

Ethnobiology is a rapidly growing field gaining prominence in global discussions about the future of our planet [1–3] because it demonstrates the interconnectedness of our biosphere and offers solutions to global problems of social and environmental injustices [5–8]. However, one of ethnobiology’s core strengths is that it is a locally grounded discipline—both in terms of data gathering and interpretation [9–11]. With the international policy community waking up to the value of ethnobiology to address global social-ecological issues [12, 13], many ethnobiologists are often faced with the challenge of communicating the global relevance of their locally based research. How can ethnobiology retain its strength as a locally grounded discipline while scaling up to amplify its clear potential relevance at the global scale?

Ethnobiology’s place-based and context-specific focus has yielded rich descriptions of place-specific human–environment inter-relations at local scales (see [3]). However, case study research can be limited in its broader applicability when linkages are not made to social and ecological contexts at larger scales [14, 15]. The almost exclusive focus on the local context has slowed efforts aimed at demonstrating patterns at larger scales and, in consequence, eschews the discipline’s potentially global contribution (in contrast with, for example, macroecology and macroevolution research; [16]). Not surprisingly, the landmark global assessment report of the Intergovernmental Science-Policy Platform on Biodiversity and Ecosystem Services (IPBES) identified the “syntheses of Indigenous and local knowledge about the status and trends of nature” as a global knowledge gap [17]. Upscaling observations made through ethnobiological practice and understanding interactions across spatial and temporal scales is necessary to realize ethnobiology’s potential to contribute to global sustainability [3, 18]. For example, aggregating results of locally based research can provide insights and help inform more effective governmental monitoring, planning, and policy development across scales [12, 19].

We contend that to continue addressing the major global societal challenges of our time, our discipline should find ways to effectively promote the scalability of place-based ethnobiological evidence and perspectives. Without losing the importance and richness of in-depth local epistemologies, practices, and their documentation [10, 11], we believe that ethnobiology should continue to upscale case-specific insights and strive to create global knowledge that can unveil general patterns and dynamics across different ethnobiological realities [20]. This call to action follows that of other fields such as political ecology where scholars have developed rigorous comparative, geospatial, and statistical approaches to reveal global trends and patterns in systematic ways (e.g. [21–23]). Such scalability is paramount in ensuring that global discussions about the future of our planet are directly connected with on-the-ground realities of Indigenous Peoples and local communities, diverse epistemologies, and human–environment interactions [19, 24, 25].

There have been criticisms of the theoretical and hypothesis-driven shortcomings of ethnobiology since at least the 1970s [26], but the multi-faceted, inter- and transdisciplinary nature of this field complicates discussion of theory-building and comparative research [27]. Despite this, ethnobiologists have long been engaged in many globally relevant research avenues from a wide range of disciplinary homes (e.g. through the application of different ethnobotanical indices [20, 26]). Some of the areas where ethnobiological evidence has been robustly built at global scales include studies on the global distribution of plants used by humans (e.g. [28]), animal and plant domestication (e.g. [29]), the commonalities and differences between distinct systems of ethnobiological classification around the world (e.g. [30]), and the co-evolutionary dynamics between biological, cultural, and linguistic diversity (e.g. [31]).

In this article, we set the ground for “global ethnobiologies” by presenting conceptual and methodological approaches to continue building relevance “from the ground up” in ways that explicitly embrace and honour contextual complexities, and at the same time incorporate a deliberate global outlook. We highlight four pathways that can bridge the gap between local contexts and global realities. Theoretically, our analysis builds on the notion of glocalization in contemporary social theory [32]. The concept of “glocality” encompasses the interpenetration of global and local spheres [33], which is relevant to the spaces where ethnobiology operates [8, 18, 34]. Drawing on this theoretical body, we provide examples of ethnobiological research and projects that are leading the way in promoting local-to-global connections, as well as a thorough description of some of the key epistemic, ethical, and methodological challenges involved in such upscaling. While our research is explicitly grounded in the discipline of ethnobiology [35], our analysis is also relevant to other disciplines conducted within explicit place-based foci, such as human ecology, historical ecology, or environmental anthropology, among others (e.g. [2, 36, 37]).

## Opportunities for developing global ethnobiologies

Here, we present four complementary pathways to enable scalability of place-based ethnobiological data and research. Each pathway is relevant to different sets of ethnobiological inquiries, but all are based on the aggregation across scales of place-based, culturally grounded research data.

### Scaling up by connecting through dialogue on-the-ground realities and global research and decision-making

While Indigenous Peoples and local communities worldwide are incredibly diverse, representing unique historical, cultural, and ecological contexts, many also experience common threats and challenges to their livelihoods and well-being, often relating, directly or indirectly, to ongoing settler and extractive colonialism and environmental loss and deterioration (e.g. [38, 39]). When locally based ethnobiological research is shared through scientific and popular publications, documentaries, artistic interventions, global dialogues, and science-policy platforms with researchers and communities globally, its value is multiplied manyfold. For instance, sharing perspectives through these fora is essential to developing effective and equitable policy responses to the climate crisis and the erosion of biocultural diversity [40]. Dialogic approaches like art, literature, and other forms of knowledge mobilization can be facilitated through numerous different organizations and institutions operating at the global scale (e.g. [17, 41, 42]).

Global dialogues, bringing together scientists and Indigenous and local knowledge holders, hold promise for connecting different knowledge systems across spatial and temporal scales and for identifying general patterns and commonalities across different biocultural contexts (e.g. [40, 41]). This moves toward creating broader policy-relevant knowledge that is also context sensitive and can enhance the ability of communities to thrive [43, 44]. The multiple evidence-based approach, for example, is often used as a method for multi-scale triangulation [41, 45]. Such research strategies require actively navigating convergences and divergences across knowledge systems, and putting a strong emphasis on the complementarities, synergies, and cross-fertilization of information derived from different scales and methods [24]. These processes usually involve multiple engagements between ethnobiologists and Indigenous and local knowledge holders working together to provide a comprehensive evidence base that can be used to inform global governance processes in meaningful ways [24, 46]. For instance, by compiling local testimonies, oral histories, articulating local governance structures, and synthesizing in-depth narratives from case studies from all over the world, numerous works have provided qualitative evidence to inform policy-makers of the impacts of environmental change or pollution on the lifeways of Indigenous Peoples and local communities (e.g. [47, 48]).

### Scaling up by aggregating published ethnobiological data

Aggregation of published data to build thematic databases, whether analysed using descriptive statistics or more complex meta-analyses, can also promote the global relevance of Indigenous and local knowledge (e.g. [15]). Compilations of published data linking cultural and environmental diversity have resulted in the creation of open-access online databases such as the recently compiled global overview of culturally important species [13], the Ethno-ornithology World Atlas (EWA; www.ewatlas.net), and global conservation surveys of all palm species with documented ethnobotanical uses [49]. As a case in point, the data collected in D-PLACE, a Database of Places, Language, Culture, and Environment, a corpus of information for over 1400 human societies [50], highlights studies at the intersection of environment and religious diversity [51], the global geography of human subsistence [52], or drivers of global variation in land ownership [53]. Such datasets compile place-based, georeferenced evidence with rigorous ground-truthing (sensu [54]). They harmonize data from distinct sources that use diverse vocabulary, focus on different temporal and spatial scales, and explicitly account for spatial and historical dependencies between groups. The creation and use of cross-cultural databases in anthropology [50, 55, 56], along with current efforts to underscore the potential of quantitative methods in ethnobiology [20], can guide and inspire new research for global ethnobiology complementing necessary inductive approaches at all scales.

There is an increasing number of ethnobiological studies relying on published data to reveal commonalities and overarching patterns in peoples’ experiences across broad regional scales [28, 57, 58]. For example, collaborative research has provided the first-ever global analysis of biocultural approaches to pollinator conservation practised by Indigenous Peoples and local communities worldwide [59], and a global overview of ethnothalassic interactions between coastal communities and marine ecosystems [60]. Other examples of such ethnobiological research describe the global impacts of climate change among subsistence-oriented societies [61], or the impact of under-documentation of Indigenous and local knowledge on policy-making [62, 63]. While building and using global databases is not straightforward, and awareness of associated issues should guide their use (see an analysis of limitations in [55, 56]), they can inspire avenues for bringing together local case studies to make intercultural inferences in globally relevant, yet ethnographically grounded, ways.

### Scaling up through multi-sited and cross-cultural research design

Scaling up from local to regional and global scales is also possible through multi-sited research design, comparing observations and data collected from across field sites using paired research protocols [64]. Multi-sited studies are linked by common research questions, data collection methods, and analyses (e.g. [65]). Multi-sited research is routinely mobilized to understand the diversity and consistency of various aspects of ethnobiological systems across time and space and to infer the processes leading to temporal or spatial change (e.g. [66, 67]). For example, inter-regional specific studies can highlight cross-cultural similarities based on criteria such as age, gender, profession, and wealth (e.g. [68–70]). Syntheses of floristic, linguistic, and political contexts of contiguous social or ethnolinguistic communities are also conducted (e.g. [71, 72]), as well as studies on the evolution and adaptation of medicinal plant uses in the context of migrations (e.g. [73–75]). Cross-cultural research adds depth and richness to ethnobiological data and contributes to hypothesis testing and theory building within the discipline [76, 77].

We envision an expansion of such approaches by designing multi-sited studies across larger spatial scales. While designing multi-sited studies across regional or global scales requires substantial coordination and funding, data collection and analyses should be facilitated by use of a consistent, data sovereign, and thoughtfully designed protocols. Protocols developed for cross-cultural research are a compelling pathway for bringing ethnobiology into global resolutions (e.g. [78, 79]). This is because such protocols are often based on pre-defined broad domains or groupings that are comparable cross-culturally, but based on locally adapted and culturally specific categories and metrics [80]. While multi-sited studies have so far mostly used concepts and classifications largely based on western scientific domains of inquiry, multi-sited research projects led entirely by Indigenous communities are emerging (e.g. Kogi territorial diagnosis “Shikwakala”; https://www.tchendukua.org/shikwakala). Moreover, the opportunity to involve community members as co-researchers in these studies can bolster the establishment of horizontal cooperation and equity conditions [81], which may further ensure that the scaling up process is accountable, and does not unnecessarily reduce or decontextualize culturally specific knowledge.

### Scaling up by applying global-scale geospatial analyses

Ethnobiologists are increasingly partnering with conservation biologists and geographers to map and characterize the different ecological values of Indigenous Peoples and local communities’ land-based stewardship from local to global levels (e.g. [82, 83]). While the case for the global significance of Indigenous stewardship has been developed by Indigenous scholars and philosophers for decades, if not longer (e.g. [84, 85]), geospatial analysis is increasingly used to visualize and quantify the contributions of Indigenous Peoples and local communities to global biodiversity conservation [83, 86, 87]. These advances, based on the best available evidence, are being employed to support current policy discussions around the role of Indigenous Peoples in global biodiversity maintenance [17, 88]. In particular, these data are being used to support Indigenous Peoples’ advocacy in international negotiations leading to the newly established Kunming-Montreal Global Biodiversity Framework of the Convention on Biological Diversity [19, 89].

Such approaches have so far demonstrated that Indigenous Peoples manage or have tenure rights over more than a quarter of the world’s terrestrial surface, intersecting with at least 37% of all remaining natural lands across the Earth [90], and 36% of the world’s most ecologically intact forests [91]. Recent geospatial analyses of mammal diversity across mapped Indigenous Peoples’ lands globally have determined that at least 60% of all the terrestrial mammal species for which there is reliable habitat data depend on these territories [92]), and that they account for 30% of the global non-human primate distribution range [93]. Although Indigenous Peoples’ lands are by no means immune to biodiversity loss [23], several global studies show that these territories host more species than equivalent protected areas and are at least equally effective in buffering against deforestation [94–96]. All these studies have led to increasing appreciation of, and interest towards, the specific practices, efforts, and knowledge systems that underpin the ecological integrity of these territories at the global level [25, 90].

## Challenges in global ethnobiological endeavours

Given that ethnobiological knowledge is often grounded within specific temporal and spatial scales, scaling up these cultural practices, to become regionally and globally relevant, risks alienating, marginalizing, or muting local insights, challenges, and perspectives. In this section, we consider some of the main tensions that have made scalability from local to global applications a challenge for ethnobiology. We also illustrate recommendations to navigate such tensions with three examples out of a growing number of ongoing projects and initiatives that are enhancing the regional and global reach of the discipline in iterative and reflexive ways (Table 1).

**Table 1:** Three examples of ethnobiological projects that illustrate best practices and potential challenges of scaling up from local to global

Project	Indigenous Peoples’ Food Systems for Health—a global approach	Building up grassroots collaborative networks across the Pacific	Identifying global commonalities and patterns in the forage-related knowledge of pastoralist communities
Aspirations	To improve the nutritional status, health, and well-being of Indigenous Peoples worldwide To document the close relationship between food and ecosystem sustainability for maintaining Indigenous Peoples’ food systems worldwide	To give global attention to sustainability, effectiveness, and revitalization of biocultural mariculture traditions, and to understand how these traditions are situated within current local, regional, and global social-ecological contexts	To globally synthetize pastoralists and herders’ central, but understudied knowledge on forage plant-livestock-herder interactions, and its use in herd and pasture management To identify global principles and widespread commonalities of this space-based and context-dependent knowledge to help develop more adequate national, regional and global-level policies, and contribute to the upcoming International Year of Rangelands and Pastoralists in 2026
Best practices for upscaling	Connection with FAO Local-to-global dialogues across knowledge systems Close collaboration with Indigenous communities, and active participation by local knowledge keepers and food specialists Training and knowledge exchange across academic and local communities relating to food, nutrition, culture, and environment Support communities in advocating for spaces/places where local foods can be safely, affordably, and sustainably obtained	In-person and virtual knowledge exchanges featuring place-based contexts within a global network Creation of compelling, accessible website that highlights both local diversity and regional connectivity among traditional marine management systems Systematic analyses of diverse aquaculture systems Honouring local knowledge as part of regional network of knowledge Ensuring opportunities for cross-generational learning (e.g. elders-to-youth) Operating from foundational values and protocols shared more globally among Indigenous knowledge holders (such as reciprocity and relationality with land and ancestors; as opposed to extractive interactions)	Semi-systematic global review of white and grey literature, including documentary films with herder voices Interviews and dialogues with pastoralists from different regions to help interpret review results on cross-cultural commonalities Multi-cultural author team from some key pastoralist regions (Mongolia, Iran, Kenya, Central Europe), also including members of local communities
Challenges of upscaling	Indigenous knowledge emerges from very different epistemological contexts than Western science, which poses challenges for global aggregation Regional unevenness in existing documentation of Indigenous food systems Sufficient and sustained funding support is a critical limiting factor on ensuring the participation of Indigenous knowledge holders	Logistical and environmental costs of bringing people together from a vast region Capacity within groups to maintain linkages among groups Access to and appropriateness of using new communication technologies (e.g. videoconferencing, digital storymaps) may be misaligned with cultural or sacred forms of expression Institutional and individual barriers to operating with cultural values and protocols common to Indigenous knowledge holders (such as short funding terms, transactional university spending policies, etc.)	Small number of publications and documentaries that documented the knowledge of local communities in their original wording (cf. emic) The identification of common principles blurred by cultural and ecological diversities of pastoralism across different regions Limited understanding of most traditional knowledge holders of the regional and global level situation, because information sources available to them (e.g. TV, radio) rarely share information of forage-related pastoral knowledge
Scaling up pathways employed *	1, 2, 3	1, 2, 4	2, 1, 3
Sources	[97–99]	[100, 101]	[102]

**Fig. 1:**
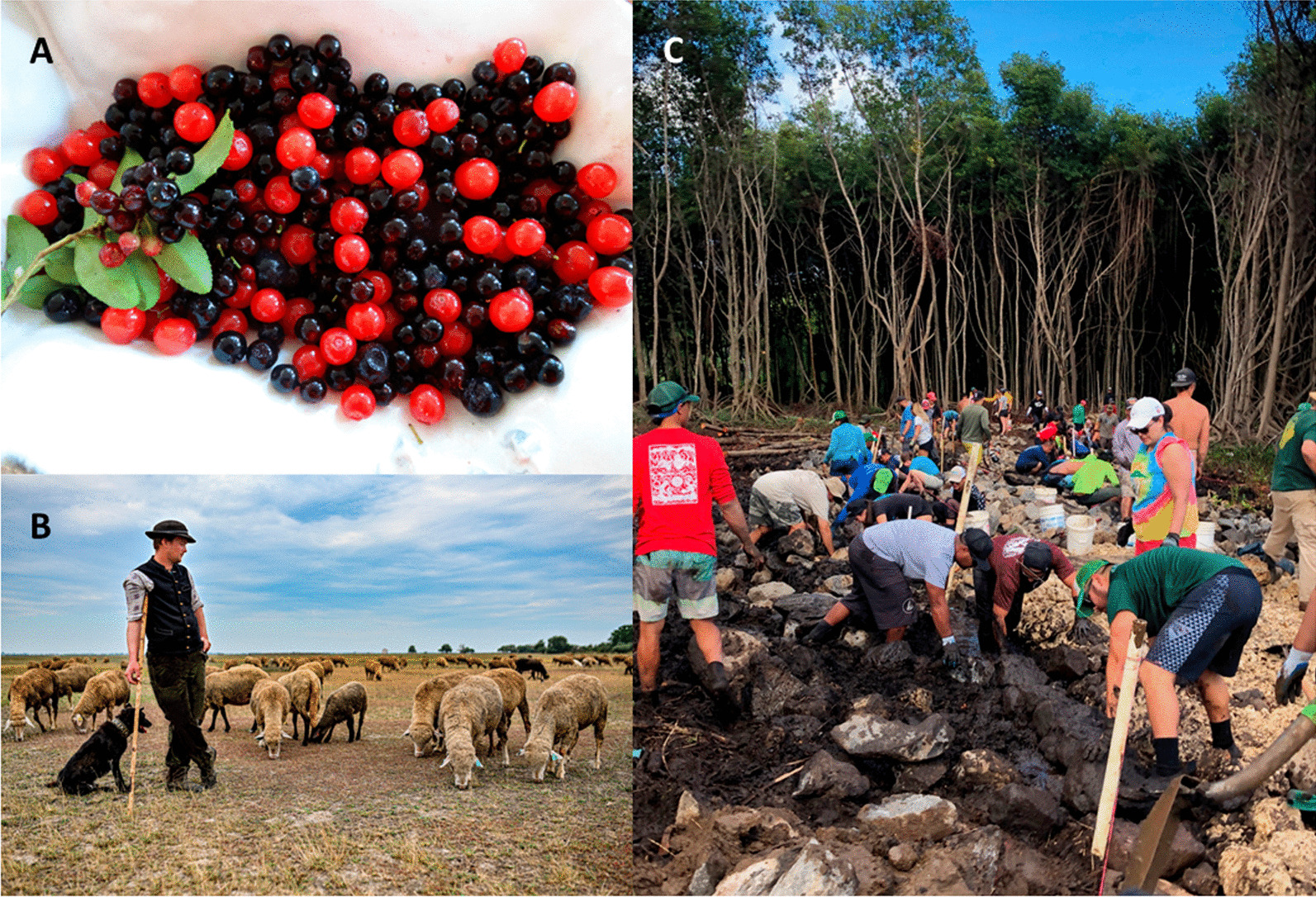
Photographs representing the three projects presented in Table 1. **A** Wild berries, such as blueberries, huckleberries and cranberries in the genus *Vaccinium*, are a nutritious food for many Indigenous Peoples. Shown here are evergreen huckleberries (*Vaccinium ovatum*), and red huckleberries, (*V. parvifolium*) from the west coast of Canada. Credit: Nancy J. Turner. **B** Indigenous knowledge holders and partner organizations participating in the Cross-Pacific Indigenous Aquaculture Collaborative Network (https://www.seagardens.net/) collectively work to restore a 100-foot segment of rock wall in a traditional loko i’a (fishpond system) on Oahu, Hawai’i in 2020, as part of a series of knowledge exchanges bringing communities together. Credit: Melissa Poe. **C** Herders in Hungary use their forage indicators in context-specific management decisions, with a variety of objectives to optimize grazing under different social and ecological circumstances. Credit: Sándor Karácsony

Note that conversations that crosscut communities are foundational to scaling up in all cases (see Fig. 1 and Additional file 1 for further details on each of these projects)

^*^ See text for a discussion of the four pathways for scaling up from local to global

A core challenge is that as projects are scaled up to provide larger, statistically significant sample sizes for comparative studies, it becomes increasingly difficult to have the project driven and steered by individual communities. That is, because western scientists in the Global North will often have the financial resources to support large-scale projects, they often assume directorial or coordinator roles with the power to control decision-making. As Wolverton et al. [103] highlight in environmental archaeology, but equally applicable to all ethnobiology, these power dynamics leave western scientists, “*to tell the story of the past for another people, thus potentially causing harm, or promoting the goals of the scientist without any real benefit for the community*”. Several ethnobiological studies have been driven by a researcher’s abstract curiosity or funding priorities at a given time rather than community interests [11]. While we strive for theoretical and methodological rigour and development in our field, any globally relevant theory or praxes should align and directly complement with calls to prioritize community-led research attuned to local interests [7]. Building sincere and lateral partnerships with Indigenous Peoples and local communities is critical to ensure that global ethnobiology is aligned with the goals and values of community (e.g. [104]). Additionally, funding agencies have an important role to play in supporting efforts to amplify the global relevance of locally based research. Providing strategic funding for interdisciplinary work on “global ethnobiologies” (e.g. through the creation of applied funding streams, framing calls for long-term interdisciplinary work, seed funding for supporting collaborative networks) will help in making ethnobiology realize its full global potential. Several research networks (e.g. Programme for Ecosystem Change and Society, Knowledge Action Networks of Future Earth) have been established to help bring place-based social-ecological research into global resolutions [36], and can offer inspiration to the broader field of ethnobiology. 

Another core challenge relates to “open data” and the question of data sovereignty [105–108]. Open-source data is beneficial for researchers doing global comparative work (e.g. Forest Data Partnership), but has come with attendant challenges to Indigenous Peoples and local communities' inherent rights and data sovereignty [108, 109]. In some cases, Indigenous data sovereignty movements are a direct response to the push for responsible open data or for the repatriation/rematriation and return of data previously extracted from community (i.e., #databack) [106]. Platforms like Mukurtu (https://mukurtu.org) make some data publicly accessible but allow for “graded access” codes to build a platform that fosters relationships of respect and trust. Similar approaches have been employed by linguists to great effect with the DELAMAN archive network (https://www.delaman.org) which has allowed great global comparative work, while also safeguarding secret, sacred, or otherwise private data. A similar centralized, graded access database for ethnobiology could be useful, but there are caveats that need to be dealt with and attenuated by ethnobiologists, namely the occasional incompatibility of “open” and “protected” data. It is important to note that access decisions should never be made by outside researchers alone, and that community input is vital to these endeavours [107–109]. The open source platform OpenTEK from the LICCI Project (https://opentek.eu/licci) is proactively using Traditional Knowledge labels to deal with these issues (see also http://digitalsqewlets.ca/traditional-knowledge_connaissances_traditionnelles-eng.php). A number of ethnobiologists managing biocultural collections at different institutions are working to deal with this issue of open data and data sovereignty as well (e.g. EWA https://ewatlas.net/ with ethno-ornithological data; [7, 107]). Developing mechanisms for community oversight of data collection, management, accessioning, and use, is a constantly moving target requiring deep collaboration and ongoing consent—ethnobiologists should be mindful that community members are often under-sourced and overworked and should be supported for taking on oversight roles [108]. For example, establishing community review boards or working with community members to develop guidelines for data sharing and use is a viable option for some projects, but should come with extensive resources to support those community members [109] and enable their participation in research projects [81]. It is also important to acknowledge that not all communities will always be interested in leading or engaging in such efforts, as they might have other more pressing social issues to deal with. Without question, any globally relevant comparative ethnobiological data management tools must adhere to basic data sovereignty principles (e.g. Ownership, Control, Access, Possession or OCAP®) including ethical principles of redress, compensation where appropriate, and recognition of the intellectual property rights of all Indigenous and local communities [108, 110].

Finally, although quantitative ethnobiology represents a fundamental aspect of the discipline, studies on human–environment interactions are largely based on qualitative data collection and analysis [111, 112]. While quantitative studies offer an enormous potential in informing policy [23], qualitative approaches often better capture the holistic aspects of Indigenous and local knowledge systems that are difficult to articulate with reductionist quantitative science [113, 114]. However, in qualitative studies, sometimes detailed descriptions of research designs and methodologies are insufficient for later comparison [115]. The methodological diversity that has always characterized the field of ethnobiology poses in itself a challenge for the global scalability of ethnobiological evidence, given that insights from a particular place and context cannot always be exported or scaled up [36]. Even when descriptions are sufficient, divergences on how data were collected, reported or interpreted can hamper large-scale comparison and quantification (e.g. [44]). On the other hand, the search for quantifiable, universal formats that are amenable to integration into global datasets come with some methodological challenges. These include the potential to crowd out empirical and contextually rich case studies, ignoring outliers, erasing epistemic, cultural, and geographic differences, and flattening vital heterogeneity and complexity [44, 116]. Importantly, all these different approaches should be seen as complementary, while acknowledging that both quantitative and qualitative data can work in synergistic and mutually enriching ways [111, 117, 118].

## Conclusions

There are many recent examples in which large groups of ethnobiological researchers (often dispersed across institutions and world regions) have self-organized to pool intellectual and material resources in pursuit of common research goals, upscaling ethnobiological evidence and data to address global social-ecological issues (Table 1). While some of these approaches have a long pedigree in ethnobiological science (e.g. cross-cultural research; [67, 68, 119]), not all of them have been mainstreamed as potential pathways to solidify the global relevance of ethnobiology. Such global assessments should be constructed from the ground up, by collaborating with Indigenous Peoples and local communities, and by utilizing existing case studies within local and regional contexts and grounding their multi-sited, cross-cultural analyses in rich contextual insights emerging from primary field-based evidence [59, 120, 121]. It is paramount to ensure that the growth of global, multi-sited ethnobiology does neither replace nor co-opt locally grounded research, and does not sacrifice depth for breadth [122]. When weighing the pros and cons of such global endeavours, we conclude that in many cases of scaling up, the risks are worth the potential contributions, when ethical considerations are carefully examined and communities are involved at all levels. In other words, ethnobiology thrives when the process of scaling up builds on, and is informed by, rich place-based research. There is evidence that the foundation of most—if not all—ethical and powerful ethnobiological studies start and end with meaningful cross-cultural discussions and local-to-global exchanges, which offer an enormous potential for giving global recognition and visibility to Indigenous and local knowledge holders [1, 3, 8].

Developing global overviews allows for deeper empirical understanding of drivers and pathways of change (e.g. [38, 123]). Such overviews can lead to more effective policies and approaches to address them, whether this might mean the development of specific legislation and regulations, or support of particular conservation, restoration, or environmental management practices (e.g. [124–127]). Some of these approaches draw inspiration from middle range theories (i.e., generalizations that describe causal mechanisms within certain contextual bonds; see [128]), and theory on processes of glocalization (i.e., simultaneous occurrence of both universalizing and particularizing tendencies in contemporary ethnobiological systems; [33]). The methodological approaches highlighted in this paper offer a strong potential for identifying global patterns in ethnobiological systems and praxes, improving causal inferences in ethnobiology, and expanding global knowledge of the factors, pathways, and mechanisms associated with different social-ecological processes around the world [25]. There is also value in the fact that methods developed in specific local contexts can be adopted by other communities to overcome parallel challenges (e.g. [129]).

Ethnobiologists are ideally suited to make linkages across scales in socially and culturally appropriate ways [130, 131], given the discipline’s strategic position at the confluence of different disciplines and ways of knowing [47, 132, 133]. Ethnobiologists usually have the language, interdisciplinary training, and cross-cultural perspective to do this in ways that other scholars may not [10, 134]. However, in making ethnobiology globally relevant, ethnobiologists must continually affirm their responsibilities to the Indigenous Peoples and local communities they work with, for, or are part of [135]. This encompasses a range of ethical concerns such as data sovereignty, appropriately acknowledging knowledge sources and data access [7]. It also is grounded on the discipline’s obligation to produce the most robust data possible so that it can stand up in court-of-law or as the foundation for better policies for both people and nature [8, 136, 137].

### Supplementary Information

**Additional file 1**. Full description of three examples of global ethnobiological research.

## Data Availability

Not applicable. Three examples illustrating our propositions are presented in detail as Additional file 1.

## Abbreviations

EWA: Ethno-ornithology world atlas
IPBES: Intergovernmental science-policy platform on biodiversity and ecosystem services
OCAP®: First nations principles of ownership, control, access and possession
