# Supplementary material for: The global relevance of locally grounded ethnobiology
Source: J Ethnobiol Ethnomed. 2024 May 18;20:53. doi: 10.1186/s13002-024-00693-w (PMC11102124; doi:10.1186/s13002-024-00693-w)
Supplement: Supplementary file 1 — Additional file 1. Full description of three examples of global ethnobiological research. [file 13002_2024_693_MOESM1_ESM.docx]

**SUPPLEMENTARY MATERIALS**

**Three examples of global ethnobiological research**

Indigenous Peoples’ Food Systems for Health – a global approach

A prime example of local-to-global ethnobiological research is the collaborative project on *Indigenous Peoples’ Food Systems for Health* – documenting the close relationship between food and ecosystem sustainability to maintain the health and well-being of Indigenous Peoples worldwide. The project had its first beginnings in the 1980s as a focused, community-based research project with ethnonutritionist Harriet V. Kuhnlein working in collaboration with the Nuxalk Nation in the Bella Coola Valley on the central coast of British Columbia, Canada [1-4]. The original research on Nuxalk food and nutrition was expanded to other, related ethnonutritional research in British Columbia [5, 6] and elsewhere in Canada to understand the cultural and nutritional benefits of Indigenous Peoples food systems, and the concomitant risks of changing them [7-8].

Leading from this research, and launched through the Centre for Indigenous Peoples’ Nutrition and Environment (CINE) at McGill University, the international *Food Systems for Health* program emerged. Kuhnlein partnered with Dene National Chief Bill Erasmus and many academic and community colleagues, both Indigenous and non-Indigenous. The program was supported by Canadian funding agencies and promoted by the Food and Agriculture Organization of the United Nations (FAO). It focused, altogether, on the knowledge and experiences of 12 rural groups in eight countries, not only documenting the traditional food systems of people in these diverse communities, but also determining how their diets have changed in recent years [9,10]. Finally, locally relevant interventions were generated with participatory research to help inform and guide people’s dietary choices to increase use of their local dietary biodiversity. Over the course of this 10-year multifaceted program, the researchers and community representatives met together in different locations to exchange experiences, findings, and ideas. This extensive research program released two major volumes through FAO, available for downloading, in whole or in part, by anyone interested in the work or in health, nutrition and environment from a multidisciplinary perspective [6, 10]. This work has been instrumental in the creation of the FAO Global-Hub on Indigenous Peoples’ Food Systems that is especially valuable for planners, policy formulators, and decision-makers at local, national, and international levels [11].

Building up grassroots collaborative networks across the Pacific

Creating and supporting cross-community collaborative networks is a potent way for ethnobiologists to highlight and uphold the global relevance of ethnobiological knowledge. A powerful example of this is the local and regional networks that developed across the Pacific to facilitate reconnections with traditional marine stewardship practices. Some of these networks are entirely community-based (e.g., <https://kuahawaii.org/>; <https://www.ebiilsociety.org>); while others are academic-local partnerships that more explicitly integrate Western and traditional knowledge at the local and regional levels (<https://www.clamgarden.com/>), and NGOs led advocacy-oriented networks where multiple actors converge ([www.mednatureculture.org](http://www.mednatureculture.org) ). In 2019, with the formation of the Cross-Pacific Indigenous Aquaculture (IA) Network (<https://indigenousaquaculture.org/>), previously disparate community groups, researchers, educators, and managers across the Pacific began coming together to celebrate and give global attention to sustainability, effectiveness, and revitalization of biocultural mariculture traditions, and to understand how these traditions are situated within current local, regional, and global social-ecological contexts [12]. Among the IA Network’s goals are to facilitate shared learning and solidarity across the Pacific-basin wide communities of practice. Shared learning is facilitated in part through in-person and virtual knowledge exchanges and fellowships for Indigenous students, as well as systematic analyses of diverse Indigenous aquaculture systems.

Comparison of Indigenous practices from twenty-two unique place-based contexts that span a globalized geography across the Pacific Ocean was made possible through a consistent analytical framework organized around topics such as: ancestral connections; geographic and temporal extents; biophysical manipulations; target species; ceremony and stewardship; and their current status [13]. While important differences exist, these cultural-ecosystems share some key characteristics that ethnobiological research helps to illuminate with global implications, namely: Indigenous aquaculture systems have been designed in light of local ecological conditions and honed over hundreds of years of observation and experimentation by the people who have created and maintained them (i.e., Indigenous science); their design and human modifications yield greater biological diversity; and these diverse harvest portfolios contribute to resilience of marine food systems [13]. Additional inquiry also shows the social roles played by collective caretaking of ancestral aquacultures systems, for example: building community connections, enabling knowledge transfer across generations, and healing from historical traumas such as colonialism and forced assimilation [14,15]. Beyond the exemplary cross-scale geography of this network and constituent systems (which also includes cross temporal, generational, and institutional scales), the Indigenous Aquaculture network reflect best ethnobiological practices by integrating and honouring ILK with the perspectives of Western scientists and other researchers from diverse regions and disciplines – with the goal of supporting local food security and sovereignty, heritage reconnections, and local policy initiatives.

Identifying global commonalities and patterns in the forage-related knowledge of pastoralist communities

‘Classical’ scientific reviews can also be useful to build global relevance from local ethnobiological understandings. Sharifian and colleagues [16] prepared a global review that aimed to synthetize pastoralists and herders’ central, but understudied knowledge on plant-livestock-herder interactions, with the objective to identify global principles, widespread commonalities. It is widely acknowledged that traditional herders are knowledgeable about their pastures and livestock [17], however, ecologically detailed datasets are still limited. The review built up a database (open data, available online) on traditional ecological knowledge of herders. Authors searched for 1) indicators pastoralists use to describe forage plants of their pastures, 2) how forage knowledge and forage indicators are used in pasture management, and 3) what herders know about plant-livestock interactions. They aggregated and harmonized data from very distinct sources that used diverse vocabularies. For instance, not only scientific papers (382 peer-reviewed articles) but other types of knowledge sources such as documentaries and films (18) were used where herders directly talk about forage plants. Additionally, scientists and experts were approached through emails sent to global fora and personal emails (260 persons) to find additional peer-reviewed papers and relevant grey literature and films, because keywords and search strings were often not efficient enough to find the relevant sources. The review also had a collaborative part where knowledgeable herders living in diverse social-ecological environments (Iran, Mongolia, Kenya, Hungary, and Poland) were interviewed to discuss review findings and understand complex social-ecological contexts. Three of these traditional herders were invited as co-authors of this review, besides the ten scientists from different nationalities, gender, age, and ethnicities.

The review found that for all herder communities, forages have relative values which depend much on season and livestock type [18]. Herders argued that they learn from both their animals and pastures [19]. Herders use their forage indicators in context-specific management decisions, with a variety of objectives to optimize grazing [20]. Ten global principles were identified, including, among others, a livestock-centered perspective of forages, close monitoring and targeted pasturing of various (preferred or avoided) forage species, the use of different livestock types, and well-planned spatial movements at multiple spatial and temporal scales to optimize the utilization of available plant resources. The review concluded that although herders vary greatly across the globe [17], the character and use of their traditional forage-related knowledge do seem to have striking commonalities. Understanding these commonalities may help the local-to-global-level understanding of the locally specific pastoral systems, support bottom-up pastoral initiatives and policy discussions on sustainable land management.

References

[1] Kuhnlein HV. Change in the Use of Traditional Food by the Nuxalk Native People of British Columbia. Ecol. Food. Nutrit. 1992;27(3-4): 259–82.

[2] Kuhnlein HV. How Ethnobiology Can Contribute to Food Security. J Ethnobiol. 2014;34(1):12-27. doi: 10.2993/0278-0771-34.1.12

[3] Lepofsky D, Turner NJ, Kuhnlein HV. Determining the availability of traditional wild plant foods: an example of Nuxalk foods, Bella Coola, B.C. Ecol. Food. Nutrit. 1985;16:223-241.

[4] Nuxalk Food and Nutrition Program. Nuxalk Food and Nutrition Handbook. Nuxalk Nation, Bella Coola. 1984.

[5] Kuhnlein HV, Moody SA Evaluation of the Nuxalk food and nutrition program: Traditional food use by a native Indian group in Canada. J Nutrit. Educ. 1989; 21(3):127-132.

[6] Kuhnlein HV, Erasmus B, Spigelski D, Burlingame B, editors. Indigenous Peoples’ Food Systems for Health: Interventions for Health Promotion and Policy. Montreal, QC: Centre for Indigenous Peoples’ Nutrition and Environment, McGill University; Rome, Italy: Food and Agriculture Organization of the United Nations. 2013.

[7] Kuhnlein HV, Receveur O, Muir DCG, Chan HM, Soueida R. Arctic indigenous women consume greater than acceptable levels of organochlorines. J Nutr 1995;125:2501–2510.

[8] Kuhnlein HV, Chan HM. Environment and contaminants in traditional food systems of northern indigenous peoples. Annu Rev Nutr. 2000;20:595–626.

[9] Kuhnlein HV, Turner, NJ. Cow-parsnip (Heracleum lanatum): An Indigenous Vegetable of Native People of Northwestern North America. J Ethnobiol. 1987;6(2):309-24.

[10] Kuhnlein HV, Receveur O, Soueida R, Egeland GM (2004) Arctic Indigenous Peoples experience the nutrition transition with changing dietary patterns and obesity. Journal of Nutrition 134(6): 1447-1453.

[11] FAO. The White/Wiphala Paper on Indigenous Peoples' food systems. Rome. 2021. doi: 10.4060/cb4932en.

[12] Pacific Sea Garden Collective. (2022). Sea Gardens Across the Pacific: Reawakening Ancestral Mariculture Innovations. Version 1. Washington Sea Grant at the University of Washington. <https://doi.org/10.6069/ZJB9-CG30>

[13] Levy J. Diversity and Resilience of Sea Gardens Across the Pacific Ocean. Master Thesis. School of Resource and Environmental Management Faculty of Environment, Simon Fraser University. 2023.

[14] Augustine S, Dearden P. Changing paradigms in marine and coastal conservation: A case study of clam gardens in the Southern Gulf Islands, Canada. Canad. Geogr. 2014; 58(3):305-314. doi: 10.1111/cag.12084

[15] Tadlock, S. "One day you're gonna know you won't starve here": A social and cultural exploration of Coast Salish restoration of clam gardens. Master Thesis. Environmental Health, University of Washington. 2019.

[16] Sharifian A, Gantuya B, Wario HT, Kotowski MA, Barani H, Manzano P, et al. Global principles in local traditional knowledge: A review of forage plant-livestock-herder interactions. J Env Manag. 2023;328:116966. doi: 10.1016/j.jenvman.2022.116966.

[17] Manzano P, Burgas D, Cadahía L, Eronen JT, Fernández-Llamazares Á, Bencherif S, et al. Towards a holistic understanding of pastoralism. One Earth. 2021;4(5):651-65. doi: 10.1016/j.oneear.2021.04.012.

[18] Molnár, Z. “I see the grass through the mouths of my animals” – Folk indicators of pasture plants used by traditional steppe herders. J Ethnobiol. 2017;37:522-541. doi: 10.2993/0278-0771-37.3.52.

[19] Meuret M, Provenza FD. When Art and Science Meet: Integrating Knowledge of French Herders with Science of Foraging Behavior. Rangeland Ecol. Manag. 2015;68(1):1-17. doi: 10.1016/j.rama.2014.12.007.

[20] FAO, 2021. Pastoralism – Making Variability Work. FAO Animal Production and Health, Rome. doi: 10.4060/cb5855en. Paper No. 185.
